# Forming Feasibility of High-Strength and Toughness Drill Pipe Joint Based on Plastic Deformation Mechanism

**DOI:** 10.3390/ma19112261

**Published:** 2026-05-27

**Authors:** Jing Cao, Jianjun Wang, Shangyu Yang, Ning Dang, Xiangyi Ren, Nan Zhang

**Affiliations:** 1 State Key Laboratory of Oil and Gas Equipment, CNPC Tubular Goods Research Institute, Xi’an 710077, China; 2 Daqing Oilfield Oil Recovery Technology Research Institute, Daqing 163318, China

**Keywords:** high strength, high toughness, drill pipe joint, precise plastic forming, microstructure

## Abstract

In view of the severe performance requirements of high-strength drill pipe joint in deep drilling engineering, 130 ksi high-strength and toughness drill pipe joint is taken as the research object. Thermal deformation behavior and precision forming process of a new composition system joint are investigated. Wire-cut specimens (ϕ10 × 15 mm) are tested on a Gleeble-3500 machine under 900–1200 °C and 0.01–10 s^−1^, with EBSD for microstructure observation. Results show that the small- and large-angle grain boundary cooperate to obtain the mixed structure of high dislocation density, fine substructure and fine grain, which makes the phase transformation products more fine and uniform. Combined with the finite element numerical simulation and experimental verification system, the effects of billet temperature, strain rate and die temperature on the microstructure and macro properties of the joint are analyzed, and the optimal combination of parameters is determined, including 880 °C billet temperature, 20 mm/s punch speed and 300 °C die temperature. The optimization scheme aims to achieve grain refinement and flow stress control, obtain fine and uniform tempered sorbite structure with grain size grade 10.0, and also achieve the optimal comprehensive strength and toughness, which can provide a theoretical basis and technical path for the industrial production of high-performance drill pipe joints.

## 1. Introduction

Deep drilling is a direct means to obtain deep oil and gas resources. The drill pipe string bears complex tensile, compressive, bending and torsional alternating loads in the well depth of thousands of meters, and its failure mostly occurs in the threaded connection part, which is the drill pipe joint. In the context of increasing drilling depth and increasingly complex geological conditions, the requirements for torsional strength, fatigue life and hydraulic transmission efficiency of drill pipe joints are getting higher and higher [1,2,3]. Poor forming process is prone to causing defects such as micro-cracks, coarse grains and stress concentration in the drill pipe joint, which not only greatly reduces the service life of the joint and increases the failure risk, but also brings significant economic losses and engineering hidden dangers. At the same time, it needs to spend a high cost to carry out downhole drilling tool fishing, troubleshooting and spare parts replacement, which will also lead to major losses such as drilling footage scrapping and whole-well exploration investment damage in serious cases. Therefore, the strength, toughness and uniformity of the internal structure of the forming drill pipe joint directly determine the overall performance of the drill pipe [4].

In recent years, scholars at home and abroad have done a lot of research on drill pipe joints [5,6,7,8,9,10,11,12,13,14]. Zhu designed the super-high torque double shoulder pipe joint according to the deformation coordination relationship of the drill pipe tool joint under the action of torque. Without reducing the tensile strength, flexural strength and compressive strength, the torsional capacity is significantly improved, which can better meet the drilling conditions of extended reach wells [11]. Shahani carried out contact stress analysis and stress concentration factor calculation of the drill pipe joint, obtaining the effect of preload on the stress concentration factor and as a result on the fatigue life of the tool joint and the contact stress distribution on the screw threads [12]. Santus studied the fatigue resonance of drill pipe and joint. The fatigue initiation was observed in the central region of the pipe specimens, where the stress amplitude is highest, and away from the tool joints of the connection specimens, mainly because of the different outer diameters. The maximum fatigue stress concentration factors at the connections were estimated [13]. However, the above research mainly focuses on the analysis of the thread of the drill pipe joint, and rarely involves the quality analysis of the transition zone and the overall structure of the drill pipe joint.

Zhang found that the failure reason of the drill pipe joint transition zone was fatigue failure caused by compound alternating load. The microstructure, surface quality and residual stress of the step transition zone of the drill pipe joint have great influence on fatigue life. The fatigue life of the model can be greatly improved by improving the structure, improving the surface processing quality and introducing controllable residual compressive stress [14]. However, this study does not involve the optimization of the forming process of the drill pipe joint.

The forming process of drill pipe joint is essentially a controllable plastic deformation process of metal, and its deformation mechanism directly determines the microstructure, properties, dimensional accuracy and service life of the joint [15,16,17,18,19]. Upsetting is the core plastic-forming process for manufacturing drill pipe joints. The rationality of its process parameters directly affects the streamline distribution, internal defects (such as folding, cracks) and mechanical properties of forgings [20]. At present, the traditional forming process has some problems, such as the poor matching of process parameters, uncontrollable plastic deformation and so on, which can easily lead to micro-cracks, coarse grains, stress concentration and other defects in the joint, seriously affecting the safety of the deep drilling operation. The controllable plastic deformation mechanism can realize the controllability of the deformation amount, deformation rate and deformation path by accurately controlling the metal flow, stress–strain evolution and temperature field distribution in the forming process, which is the core idea to solve the forming defects of high-strength drill pipe joints. Therefore, analyzing the controllable deformation mechanism in the plastic-forming process of the 130 ksi drill pipe joint and optimizing the key process parameters can provide theoretical basis and technical support for improving the forming quality and service performance of drill pipe joint.

The traditional manufacturing of drill pipe joints mostly adopts free forging or ordinary die forging process, which has the problems of low material utilization, unstable microstructure and properties, poor product consistency and so on. It is difficult to meet the requirements of deep drilling for the high performance and high precision of components. Precision plastic-forming technology has become the key direction to break through the above bottleneck because of its advantages of near net shape, compact structure and continuous streamline. Its core is to control the evolution of microstructure by accurately controlling the plastic deformation process of metal (i.e., controlling plastic deformation), so as to improve the mechanical properties of components [21]. The traditional process design mostly relies on “trial and error method”, which has high cost and long cycle. Computer numerical simulation technology, especially the finite element method, provides a powerful tool for in-depth understanding of material flow, stress–strain field and damage evolution in the process of metal plastic forming. By establishing an accurate finite element model, the influence of key process parameters such as blank temperature, punch loading speed and die temperature on the forming quality was systematically analyzed, aiming to provide an optimized process window for the actual production and realize the manufacture of high-quality drill pipe joints.

Therefore, this paper focuses on the precise plastic-forming process of 130 ksi high-strength and high-toughness drill pipe joint, focuses on the controllable plastic deformation mechanism, and systematically optimizes the key process parameters in the forming process with the help of numerical simulation and experimental analysis, in order to provide a feasible design scheme for the stable and efficient manufacturing of high-performance drill rod joint.

## 2. Materials and Methods

### 2.1. Materials

To meet the demanding conditions of ultra-deep wells under high temperature and high pressure, a new material composition system for drill pipe connections was developed by early studies. Low-carbon design serves as an important method to enhance toughness. Reducing carbon content enables the formation of lath martensite. Compared with high-carbon twinned martensite, low-carbon lath martensite exhibits an excellent strength–toughness matching. However, the decrease in carbon content will reduce the strength of the joint. By rationally proportioning strengthening elements such as Mn, Si, and Mo in low-carbon alloy steel and adding appropriate amounts of microalloying elements like Nb and V, the synergistic effects of solid solution strengthening, precipitation strengthening, and grain refinement strengthening were achieved, while the material’s tempering stability was enhanced; this significantly improved the steel’s strength and toughness. The quenching and tempering process of the new drill pipe joint steel material is held at 890 °C for 50 min quenching, then held at 450 °C for 120 min and tempering. The steel meets the performance requirements of ultra-deep wells by achieving a yield strength ≥ 1024 MPa and a longitudinal impact energy ≥ 116 J at −20 °C. The specific material composition is shown in Table 1.

### 2.2. Research Methodology

A study was conducted on the thermal deformation behavior of new materials for high-strength and toughness drill pipe joints, using the wire cutting method to make cylindrical shapes of ϕ10 × 15 mm, and conducting thermal compression tests on the Gleeble-3500 thermal simulation testing machine, the manufacturer is Dynamic Systems Inc., located in Poestenkill, United States. Three parallel tests were repeated under each temperature and strain rate combination condition. After removing abnormal samples with large dispersion, the average test value was taken as the final rheological stress data. Graphite sheets are used for lubrication to reduce friction, and the residual friction error is small and acceptable for analyzing thermal deformation behavior. The thermodynamic properties of the 0.08C drill pipe joint steel were analyzed at temperatures of 900 °C, 1000 °C, 1100 °C, 1200 °C, and strain rates of 0.01 s^−1^, 0.1 s^−1^, 1 s^−1^, and 10 s^−1^. The deformation amount is 60%, and quenching treatment is carried out after deformation to maintain the original structure.

After the thermal simulation compression test, the specimen was sectioned along the deformation direction, and the microstructure of the longitudinal section was observed. The specimen was then mechanically ground and polished step by step using sandpaper of grades 80#, 180#, 400#, 800#, 1200#, 2000#, and 5000#. Each grade was applied for 3–5 min to smooth out the scratches from the previous grade. After that, it was polished with diamond polishing compound and cloth for 10 min until it reached a mirror-like finish, ensuring no scratches under the microscope. Electrolytic polishing was used to remove surface stress, using an electrolyte consisting of 10% perchloric acid and 90% anhydrous ethanol at a voltage of 20 V, current of 1.5 A, and for 15 s at a temperature of 0–5 °C. After polishing, the specimen was immediately rinsed with anhydrous ethanol and quickly dried using cold air. The sample was placed in a field emission scanning electron microscope equipped with an EBSD probe for micro-orientation analysis, the manufacturer is Oxford Instruments plc., located in Abingdon, Oxfordshire, UK. AZtecCrystal 3.3 software was used to analyze and process the grain boundaries, grain morphology, and microstructural characteristics, thereby achieving systematic characterization of the micro-crystallographic information of low-carbon steel.

## 3. Study on the Plastic Behavior of High-Strength and High-Toughness Drill Pipe Joint

### 3.1. Thermal Deformation Behavior of Drill Pipe Joint Material

**Figure 1:**
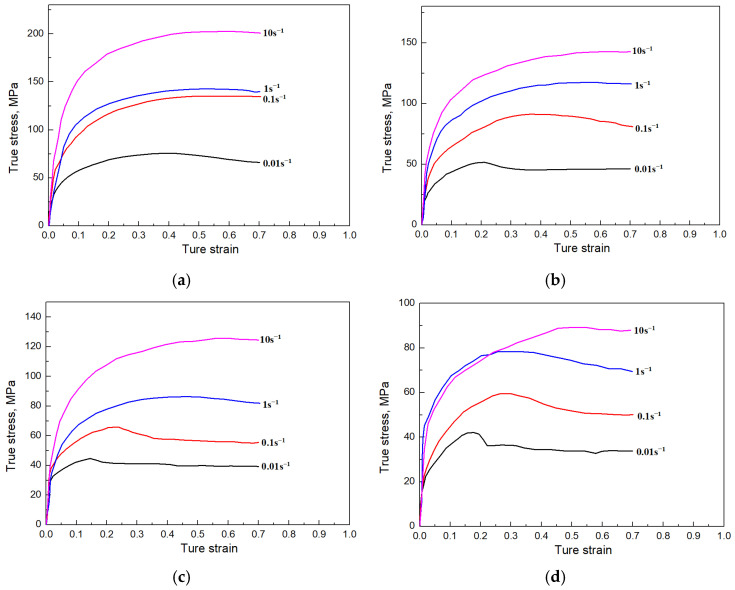
True stress–true strain at different temperatures: (**a**) 900 °C, (**b**) 1000 °C, (**c**) 1100 °C, (**d**) 1200 °C.

The stress–strain curves of the thermal deformation process at different temperatures and strain rates are shown in Figure 1; the following characteristics could be observed:

(1) The strain rate is the core factor affecting the plastic deformation resistance of 130 ksi drill pipe joint steel, and the true stress increases significantly with the increase in strain rate. At 900 °C, as shown in Figure 1a, the peak true stress at a low strain rate of 0.01 s^−1^ is about 60 MPa, while at a high strain rate of 10 s^−1^, the peak true stress exceeds 180 MPa. At 1200 °C, as shown in Figure 1d, the peak stress corresponding to 0.01 s^−1^ is about 40 MPa, and after 10 s^−1^, it rises to above 80 MPa. This is because at low strain rates, there is sufficient time for dislocation slip, dynamic recovery, and recrystallization within the material, resulting in lower deformation resistance. At high strain rates, dislocations do not have enough time to fully move and annihilate, and a large number of dislocations accumulates, leading to work hardening effects dominating, and ultimately resulting in a sharp increase in deformation resistance. At the same time, there are differences in the evolution characteristics of the curve under different strain rates; at low strain rates (0.01 s^−1^, 0.1 s^−1^), the curve slowly decreases and tends to stabilize after reaching the peak stress, reflecting the “work hardening dynamic softening” equilibrium dominated by dynamic recrystallization. At high strain rates (1 s^−1^, 10 s^−1^), the peak stress of the curve appears earlier and the decrease stage is smoother, indicating that the dynamic softening process cannot completely offset the work hardening, and the material is always in a high deformation resistance state.
(2) As the deformation temperature increases from 900 °C to 1200 °C, the overall deformation resistance of the 130 ksi drill pipe joint steel shows a significant downward trend, and the higher the temperature, the smaller the stress difference caused by strain rate. At 900 °C, the stress difference between 10 s^−1^ and 0.01 s^−1^ is about 120 MPa. At 1200 °C, the difference between the two is only about 40 MPa. This is due to the intensified atomic thermal motion at high temperatures, the activation of deformation mechanisms such as grain boundary slip and dislocation climb, the improvement in material plasticity, and the acceleration of dynamic recrystallization rate, effectively alleviating the work hardening effect. From the curve shape, at 900 °C, as shown in Figure 1a, the curves at various strain rates exhibit a “rapid rise slow decline” characteristic, with limited dynamic softening degree. At 1000–1100 °C, as shown in Figure 1b,c, the peak stress of the curve decreases significantly, and the peak strain decreases slightly, indicating that this temperature range is the active range for dynamic recrystallization of the material, and the controllability of plastic deformation is better. At 1200 °C, as shown in Figure 1d, the overall stress level of the curve is the lowest, but there are still obvious work hardening characteristics under high strain rates, indicating that even at high temperatures, too fast deformation rates can suppress the dynamic softening process and easily lead to stress concentration.
(3) The optimal temperature range is 1000–1100 °C as that balances deformation resistance and the dynamic softening effect, and the material exhibits good plastic deformation at this temperature. A medium low strain rate of 0.1~1 s^−1^ is more suitable for the controllable plastic deformation requirements of drill pipe joints. The drill pipe joint steel achieves a balance of “work hardening dynamic softening” during the forming process, ensuring deformation uniformity and dimensional accuracy, while avoiding stress concentration and forming defects.

Many literature studies have shown that during the hot deformation process, the hot deformation behavior of metal materials is mainly controlled by hot deformation parameters. Generally, the relationship between them is quantitatively expressed using three parameters, deformation temperature, strain rate, and rheological stress, namely the sine hyperbolic Arrhenius relationship [22,23,24,25,26,27,28,29], as shown in Equation (1).

(1) $$\dot{\varepsilon }=AF(\sigma )exp(-\frac{Q}{RT})$$

where $\dot{\varepsilon }$ is the strain rate (s^−1^); Q is the activation energy for thermal deformation (J/mol); R is the gas constant, taken as 8.314 J/(mol·K); T is the absolute temperature (K); A is the material constant (s^−1^); F(σ) is the stress function.

In the analysis of metal hot working, in order to more accurately describe the “flow stress” (i.e., the stress required for the material to undergo plastic deformation), researchers introduced the Zener–Hollomon parameter (Z) and found that the Z parameter has the following relationship with rheological stress, as shown in Equation (2):

(2) $$Z=\dot{\varepsilon }exp(\frac{Q}{RT})={A[sinh(a\sigma )]}^{n}$$

The scatter plots of lnσ-ln $\dot{\varepsilon }$ and σ-ln$\dot{\varepsilon }$ are drawn, and linear fitting on the above data using the least squares method is performed; *β* = 0.08775 and *n*_1_ = 7.2575. Therefore, *α* = *β*/*n*_1_ = 0.012091.

The work hardening index is calculated from Equation (3), the value is 5.395, Q is 448.54 kJ/mol, and lnA is equal to 37.89738.

(3) $$n=\mathrm{dln}\dot{\varepsilon }/\mathrm{dln}[\sinh(\alpha \sigma )]$$

Therefore, the high-temperature constitutive equation of the high-strength and high-toughness joint steel is shown in Equation (4):

(4) $$\dot{\varepsilon }=2.8749\times 10^{16}{\left[sinh(0.012091\sigma )\right]}^{5.395}exp(-448,540/RT)$$

The rheological stress at T = 1100 °C (1373 K) and $\dot{\varepsilon }$ = 1 s^−1^ under this model is calculated as 89.29 MPa. The high agreement with the peak stress of 86.3 MPa at 1100 °C and 1 s^−1^ in Table 2 confirms the accuracy of the model.

### 3.2. Controllable Plastic Deformation Mechanism

The essence of controllable plastic deformation is to control the evolution process of the internal microstructure by adjusting external process parameters (such as temperature, strain rate, deformation degree, etc.) under thermal mechanical coupling conditions, in order to obtain the ideal microstructure and ensure its mechanical properties and service reliability.

#### 3.2.1. The Influence Law of Deformation Temperature

**Figure 2:**
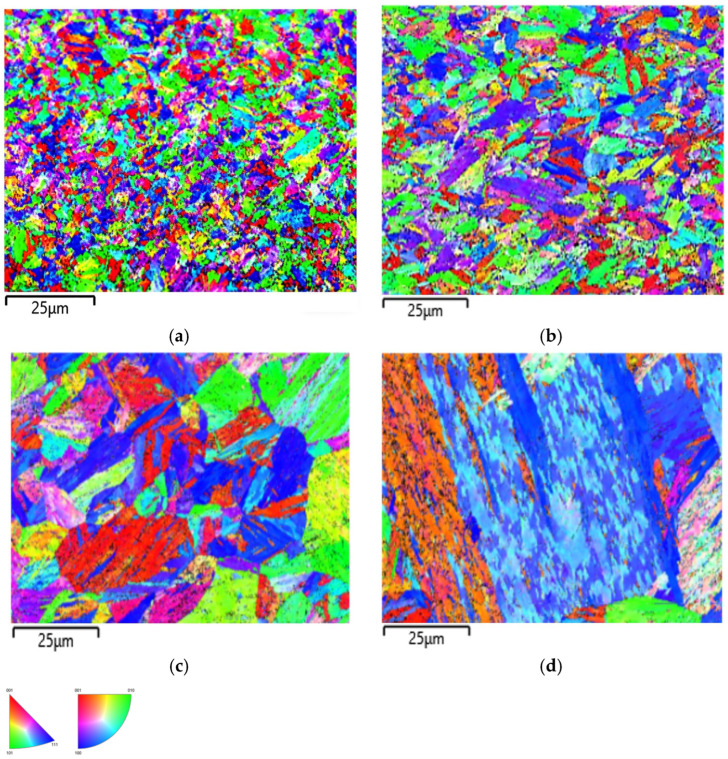
IPF diagram at different temperatures at strain rate 1 s^−1^: (**a**) 900 °C, (**b**) 1000 °C, (**c**) 1100 °C, (**d**) 1200 °C.

At a strain rate of 1 s^−1^ and 900 °C (Figure 2a), the grains exhibit a small and irregular equiaxed distribution, with an overall grain size of about 10–15 μm and no obvious preferred orientation. This is because the material has high deformation resistance at low temperatures, and although the dynamic recrystallization nucleation rate is high, the growth rate of crystal nuclei is slow, and there is no significant directional extension of grains during the deformation process.

At 1000 °C (Figure 2b), the grains are still mainly equiaxed, but a small number of slightly larger grains (about 15–20 μm) appear in local areas. The randomness of grain orientation is slightly reduced, indicating that the nucleation and growth processes of dynamic recrystallization tend to be balanced at this temperature, and some grains begin to show a slight trend of preferential growth.

At 1100 °C (Figure 2c), the grain size significantly increased (20–30 μm), the proportion of equiaxed grains decreased, and obvious grain orientation extension characteristics appeared. The aggregation area of grains with the same orientation also expanded, indicating that the growth rate of dynamic recrystallization that dominated the grain nuclei exceeded the nucleation rate at high temperatures, and grains began to preferentially grow along the deformation direction.

At 1200 °C (Figure 2d), the grains undergo severe coarsening and directional elongation, with grain sizes reaching 30–50 μm, and the vast majority of grains are distributed in strips along a single direction, with a significant increase in orientation concentration, reflecting the plastic softening of the material at high temperatures. Under the external force of a strain rate of 1 s^−1^, the grains undergo severe directional rheology along the deformation direction, and dynamic recrystallization cannot suppress grain coarsening and preferred orientation.

In the temperature range of 900~1000 °C, the grain orientation is dispersed and the texture strength is low. At this temperature, the main deformation mechanism of the material is “dislocation slip and dynamic recrystallization nucleation”. The random nucleation of a large number of small recrystallization nuclei offsets the orientation concentration caused by deformation, and the development of texture is suppressed, which is conducive to ensuring the uniformity of the microstructure after the formation of the drill pipe joint.

Within the temperature range of 1100~1200 °C, it gradually expands and exhibits a strip-like distribution, with a significant increase in texture strength. This is because the atomic diffusion ability is enhanced at high temperatures, and dislocation climb and grain boundary slip become the main deformation mechanisms. Grains undergo directional rotation and growth along the deformation direction under stress, forming strong textures. Strong texture can lead to anisotropy in the mechanical properties of drill pipe joints in different directions (such as high tensile strength along the texture direction and poor plasticity in the vertical direction), reducing the comprehensive service performance of the joint.

The optimal temperature that balances grain size and texture state is 1000 °C; at this temperature, there is no significant grain coarsening (size is less than 20 μm), the texture strength is low, the microstructure uniformity is good, and the deformation resistance is moderate. It can avoid low-temperature cracking and prevent high-temperature grain coarsening, which is highly consistent with the conclusion drawn from the analysis of the true stress–strain curve in the previous text that “1000–1100 °C is the optimal forming temperature range”.

**Figure 3:**
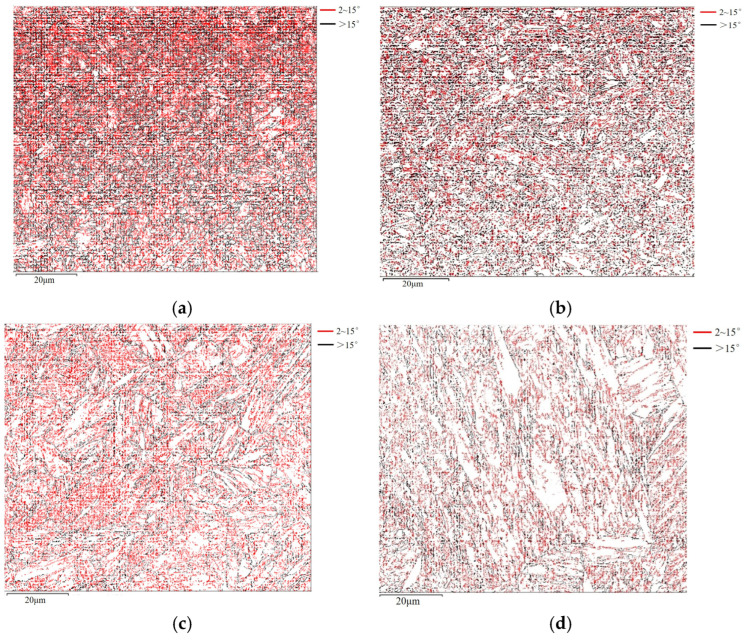
Grain boundary angle at different temperatures at strain rate 1 s^−1^: (**a**) 900 °C, (**b**) 1000 °C, (**c**) 1100 °C, (**d**) 1200 °C.

**Figure 4:**
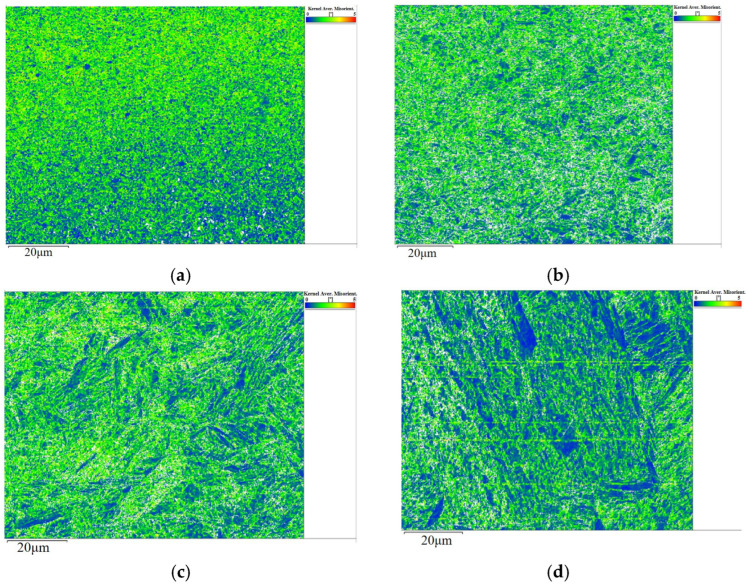
Kam diagram of different temperatures at strain rate 1 s^−1^: (**a**) 900 °C, (**b**) 1000 °C, (**c**) 1100 °C, (**d**) 1200 °C.

At the strain rate of 1 s^−1^, the grain boundary angle distribution and microstructure of 130 ksi drill pipe joint steel show regular evolution with the deformation temperature increasing from 900 °C to 1200 °C. As shown in Figure 3, the red small-angle grain boundary (2~15°) is absolutely dominant at 900 °C, the microstructure is compact and the grains have no obvious orientation characteristics; the LAGB/HAGB ratio is 1.23, the average grain size is 5.51 μm. This shows that the deformation is dominated by dynamic recovery. From the Kernel Average Mis-orientation (KAM) diagram (Figure 4), it can also be seen that the orientation difference in grains is small and evenly distributed, and the dislocation density is at a high level; the average KAM value of the material is calculated to be 1.45°. A large number of dislocations generated by dislocation slip realizes stress relaxation through the formation and migration of small-angle grain boundaries, without significant dynamic recrystallization, and the material is in a high work hardening state.

At 1000 °C, the proportion of red small-angle grain boundary decreased, and the black large-angle grain boundary (>15°) began to increase; the grains show a slight equiaxed trend, the LAGB/HAGB ratio is 1.26, and the average grain size is 3.98 μm. At this time, dynamic recrystallization starts, and some small-angle grain boundaries evolve into large-angle grain boundaries by absorbing dislocations. The dislocation density in the newly formed grains is greatly reduced, and the microstructure uniformity is slightly improved, indicating that the release of deformation energy transits from “dislocation dominated” to “grain boundary reconstruction dominated”, but the overall dislocation density remains high. At 1100 °C, the proportion of black large-angle grain boundaries increases significantly, and the grains show an obvious directional elongated morphology (arranged along the deformation direction); the average grain size is 4.12 μm. At this stage, dynamic recovery and dynamic recrystallization work together. The elongated grain morphology reflects that the deformation is still dominant. The recrystallization nucleation rate and growth rate are in dynamic balance, which is the key temperature range for both strength and plasticity. At 1200 °C, the black large-angle grain boundary becomes dominant, and the grains are significantly coarsened and directionally elongated; the average grain size is 8.45 μm, the average KAM value of the material is calculated to be 1.06°. Dynamic recrystallization is fully carried out at high temperature, the grain growth rate is accelerated, and the increase in large-angle grain boundaries is helpful to improve the plasticity and toughness of the material, but excessive coarsening may lead to strength loss. Therefore, in the temperature range of 1000~1100 °C, the synergistic effect of small-angle and large-angle grain boundary can achieve the balance of strength and plasticity, which is the ideal temperature range for the forming of drill pipe joint, which can not only ensure the bearing capacity of the joint, but also relieve the stress concentration through controllable plastic deformation.

#### 3.2.2. Influence Law of Strain Rate

**Figure 5:**
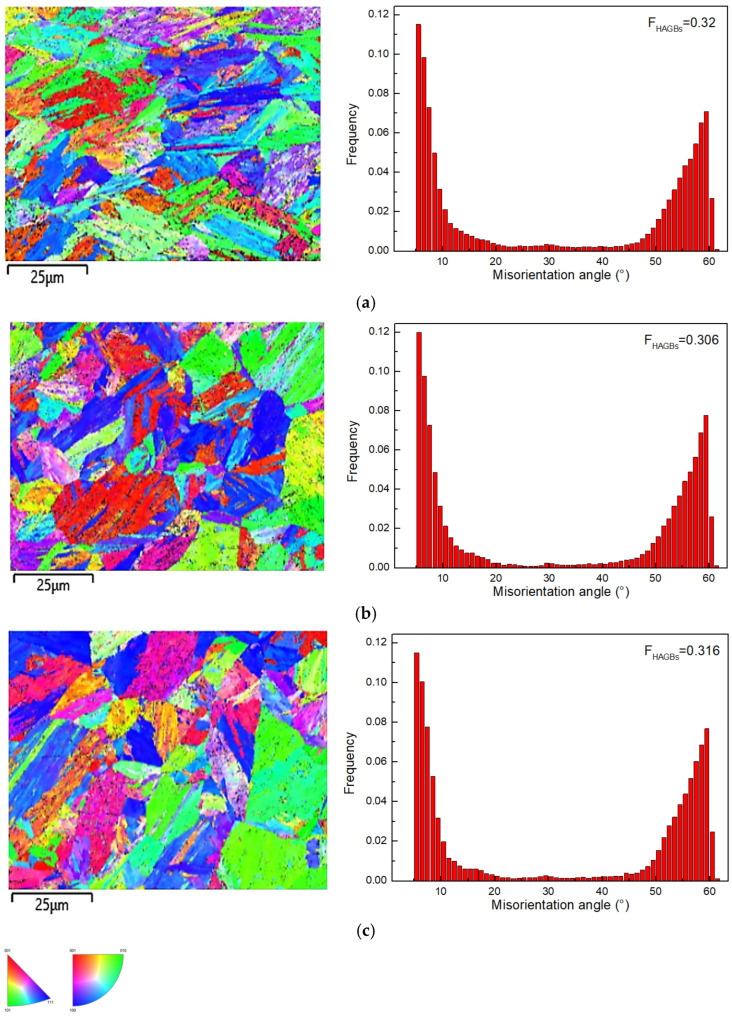
EBSD orientation diagram of different strain rates at deformation temperature of 1100 °C: (**a**) 0.1 s^−1^, (**b**) 1 s^−1^, (**c**) 10 s^−1^.

At the deformation temperature of 1100 °C, different strain rates significantly changed the microstructure characteristics of the 130 ksi drill pipe joint material. As shown in Figure 5, at the low strain rate 0.1 s^−1^, the structure presents obvious elongated flat grains, and the grains are oriented along the deformation direction. The grain boundary orientation difference is small, and the existence of a large number of small-angle grain boundaries indicates that dislocation slip and grain boundary migration are in relative equilibrium; the atomic diffusion and grain boundary migration time are more sufficient, which is conducive to maintaining high plasticity during deformation. When the strain rate is 1 s^−1^, the elongation of grain decreases, and there is a partial equiaxed trend. At the same time, the distribution of grain boundary orientation difference is more dispersed, the grain boundary density is increased, and the interweaving degree of large-angle grain boundary is increased. This indicates that the dynamic recrystallization starts, the newly formed equiaxed grains coexist with the elongated grains without complete recovery, and the microstructure uniformity is improved.

At high strain rate 10 s^−1^, the deformation energy storage accumulated rapidly, the grains are further refined, the equiaxed characteristics are more obvious, and the proportion of large-angle grain boundaries is slightly reduced to 0.316. The rapid accumulation of deformation energy at high strain rate promotes the nucleation and growth of dynamic recrystallization. Studies have shown that large-angle grain boundaries can effectively improve the low-temperature toughness of materials by hindering the propagation of micro-cracks in materials. Therefore, when the strain rate is 0.1~1 s^−1^, the mixed structure of “high dislocation density, fine substructure and fine grain” is obtained, which provides a large number of nucleation sites for subsequent transformation (such as martensite and bainite), making the transformation products finer and more uniform, and the material can take into account the synergistic improvement in strength and toughness. Therefore, the optimal temperature range and strain rate determined by thermal simulations can provide a microscopic mechanism basis for optimizing the billet temperature, punch speed, and mold preheating temperature for actual forming.

## 4. Research on Precision Forming Process of Drill Pipe Joint

In order to meet the requirements of high torsional resistance and high pressure sealing, the structure of deep drilling joint is becoming more and more complex, such as double shoulder, non-planar seal or multi-stage seal design. This puts forward higher requirements for its forming process, which requires the metal to realize accurate and uniform flow in the mold cavity to ensure that the fiber streamline of key parts (such as thread root and sealing shoulder) is complete and free of defects. Therefore, the process design must take into account the dual goals of macro forming and microstructure control.

### 4.1. Finite Element Model Establishment of Drill Pipe Joint

**Figure 6:**
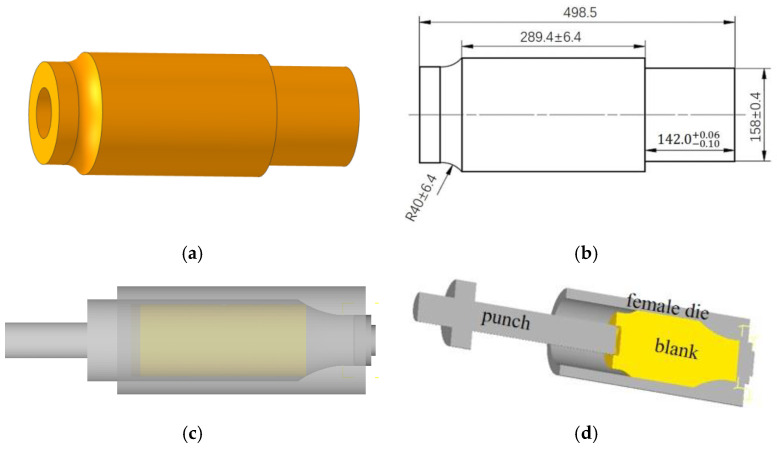
Schematic diagram of drill pipe joint part structure and assembly model: (**a**) final part, (**b**) geometric dimensions, (**c**) the first-step assembly model, (**d**) and the second-step assembly model.

The three-dimensional structure of the matching joint of the outer diameter 149.23 mm pipe body commonly used on site is constructed by using the three-dimensional modeling software. The volume of the original blank is 10,049,626 mm^3^ and the blank size is Φ 170 × 442.75 mm according to the forging equal volume method. The geometric model of the blank and the die is established, as shown in Figure 6. The blank material is 130 ksi high-strength joint steel; stress–strain curves at high temperature are shown in Figure 1, and the high-temperature constitutive model is shown in Formula (4). The thermal mechanical coupling simulation is carried out by using the metal plastic-forming simulation software. The blank is set as a plastic body and the die is set as a rigid body. The simulation process includes two stages of forming: the first stage is upsetting deformation until the blank contacts the female die, and the second stage is punch hot extrusion forming. The simulation calculation considers the heat conduction and friction between the blank and the die, the shear friction model is adopted, and the friction factor is set to 0.3. The heat exchange coefficient between the blank and the die is 11 N/(s·mm·°C); the billet and air convection coefficient is 0.02 N/(s·mm·°C) [30]. The stress field, strain field, temperature field and metal streamline in the forming process are studied. The main output variables analyzed include equivalent stress, equivalent strain, strain uniformity index, damage value and the maximum principal stress. Regarding the selection of damage models, during the hot extrusion forming process of drill pipe joints, the root of the thread and the variable cross-section area are subjected to significant tensile stress, which can easily lead to ductile cracking defects; The normalized Cockcroft and Latham damage criterion is based on the maximum principal tensile stress and equivalent plastic strain as control parameters, and is suitable for the initiation and evolution mechanism of ductile damage under high temperature and large deformation. The normalized dimensionless form facilitates the horizontal comparison of damage degree under different process parameters and has good computational stability. It has been maturely applied in the simulation of the hot forming of petroleum pipes. Therefore, this criterion is selected for damage prediction and process optimization. Here, damage values are used for the relative comparison of forming conditions, and no calibrated critical damage value for crack initiation is provided in this work. The observed reduction in damage value under the optimized parameters is an indicator of improved forming safety, rather than a quantitative prediction of fracture resistance.

Regarding the size of the drill pipe joint in Figure 6, the forming force calculated by finite element simulation is 22,500 KN, while the actual forming load required by the experiment is 23,400 KN, with an error of about 4%. The actual total length of the forged joint is 498.5 mm, and the finite element calculation shows a total length of 505.2 mm, with a dimensional accuracy of 1.3%. Therefore, the accuracy of the finite element model is within the allowable range, which can verify the reliability of the finite element model.

### 4.2. Influence of Different Forging Parameters on Joint Properties

#### 4.2.1. Influence Law of Billet Temperature

**Figure 7:**
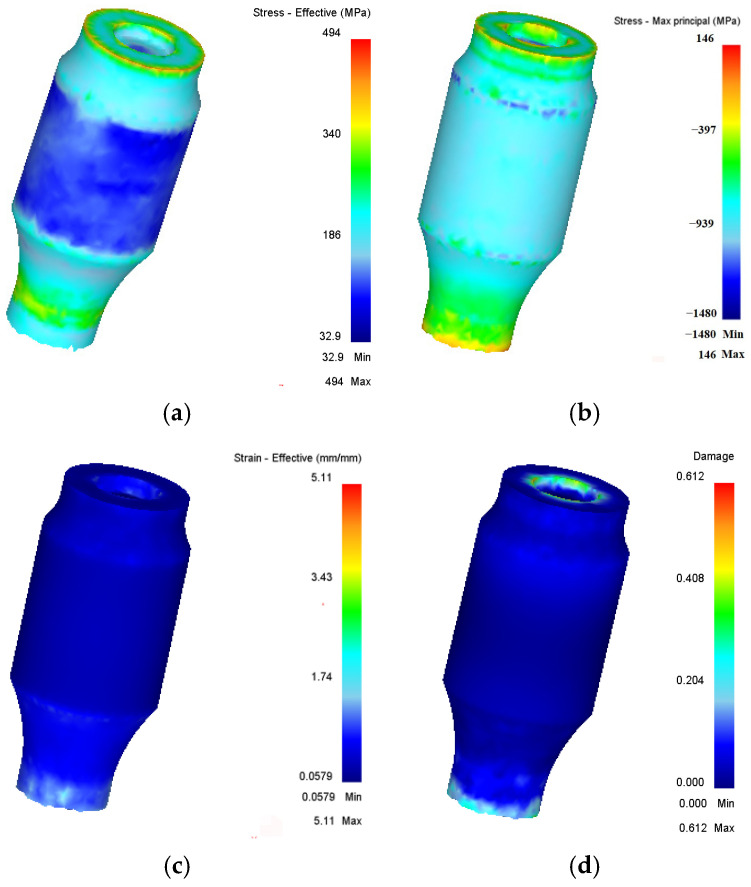
Cloud diagram of field variable distribution in the first forming stage: (**a**) equivalent stress, (**b**) maximum stress, (**c**) equivalent strain and (**d**) damage value.

When the punch loading speed is 10 mm/s and the blank temperature is 1000 °C, the equivalent stress, strain, velocity field, temperature field and loss value in the joint forming process are calculated. In the first loading stage, as shown in Figure 7, the maximum equivalent stress is 494 MPa, the maximum principal stress is 146 MPa, the strain uniformity index is 0.51 (the value is 1 − (*ε*_max_ − *ε*_avg_)/*ε*_max_), and the maximum damage value is 0.612 (normalized C&/L criterion).

**Figure 8:**
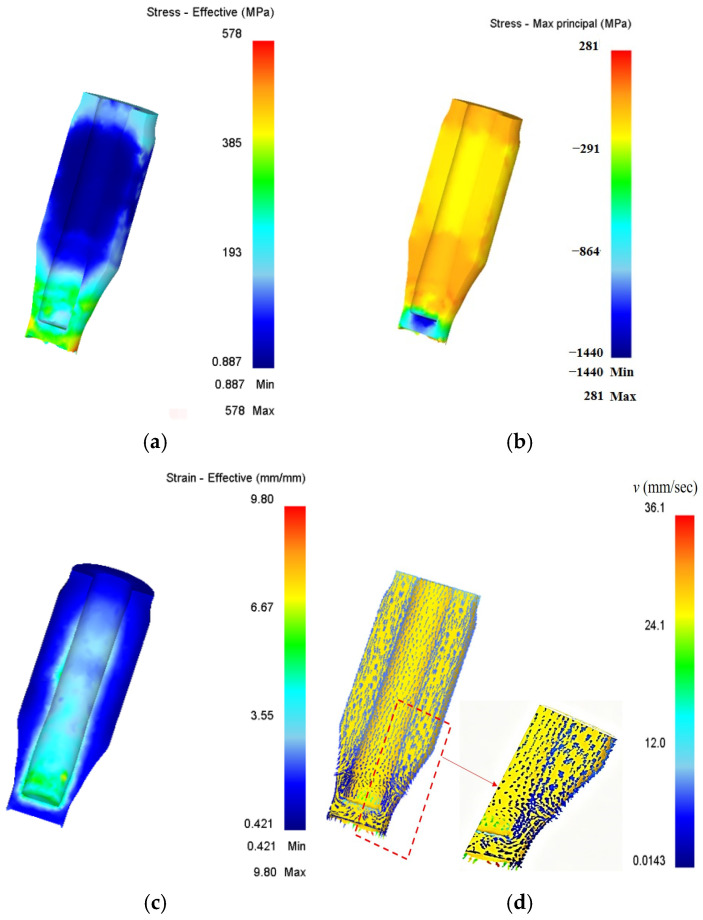
Cloud diagram of field variable distribution in the second forming stage: (**a**) equivalent stress, (**b**) maximum stress, (**c**) equivalent strain, and (**d**) velocity field.

In the second loading stage, as shown in Figure 8, the maximum equivalent stress of the joint is 578 MPa, the maximum principal stress is 281 MPa, the strain uniformity index is 0.48, and the maximum velocity value is 36.1 mm/s.

Similarly, when the billet temperature is 1100 °C and 1200 °C, the plasticity and fluidity of the material increase with the increase in temperature, and it is easier for the metal to fill the cavity. The strain uniformity index increases, indicating that the plastic deformation distribution inside the forging is more uniform, which is conducive to avoiding local strain concentration, so as to obtain a denser microstructure with more uniform mechanical properties. In addition, high temperature reduces the flow stress of the material, reduces the deformation resistance, promotes the softening mechanism such as dynamic recrystallization, and effectively inhibits the initiation and propagation of micro-cracks. Therefore, the damage value decreases significantly with the increase in billet temperature. This shows that under the condition that the equipment allows and does not produce overheating and overburning, appropriately increasing the initial forging temperature is an effective way to improve the forming quality. Combined with the above controllable plastic deformation micro-mechanism, 1100 °C should be used as the optimal hot deformation temperature in actual production.

#### 4.2.2. Influence Law of Punch Loading Speed

The effects of punch loading speeds of 1 mm/s, 10 mm/s and 20 mm/s on the forming process are studied, as shown in Table 3. In the velocity range of 1–20 mm/s, the strain uniformity index changes little. This shows that the loading speed is not the dominant factor affecting the deformation uniformity for the specific working condition of this study. The damage value shows a nonlinear relationship with the change in loading speed. When the velocity increases from 1 mm/s to 20 mm/s, the damage value decreases gradually and reaches the minimum value of 0.698 at 20 mm/s, which is the result of the competition between strain rate-strengthening effect and thermal-softening effect. The lower loading speed (1 mm/s) gives the material more sufficient recrystallization and recovery time, but the total deformation time is long; although the strain rate is high at a higher speed (greater than 20 mm/s), the deformation heat generated is too late to dissipate, resulting in an increase in the local temperature of the billet and a significant thermal-softening effect, which is beneficial to reduce the forming load and damage accumulation. By comparing the two stages, the equivalent stress and damage value in the second stage are higher than that in the first stage, indicating that the stress level and damage risk of plastic forming in this stage are higher, and the change in loading speed is more sensitive to the maximum rate and damage value. When the loading speed is 20 mm/s, the average strain rate of the joint is 1 s^−1^, which is consistent with the previous thermal simulation compression test conclusion.

#### 4.2.3. Influence Law of Mold Preheating Temperature

**Figure 9:**
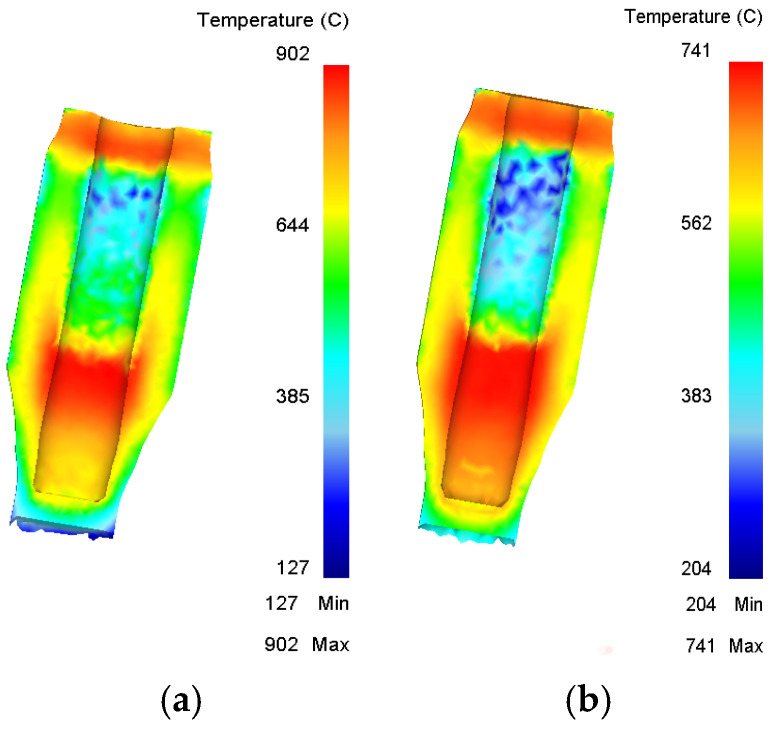
Cloud chart of temperature field distribution under different mold temperatures: (**a**) 20 °C, (**b**) 300 °C.

Mold temperature is often neglected but crucial parameter is in actual production. The simulation results of the mold at room temperature (~20 °C) and preheating to 300 °C are compared. When the billet temperature is 880 °C and the die is preheated to 300 °C, the true deformation temperature of the material remains within the target range during the forming process. The average strain rate at 20 mm/s is approximately 1 s^−1^, which is consistent with the optimal strain rate range obtained from EBSD analysis, and has the lowest damage value and good strain uniformity. Therefore, under the selected temperature and rate parameters, the equivalent stress and the maximum principal stress are greatly reduced. This is because preheating the die reduces the huge temperature difference between the blank surface and the die, as shown in Figure 9, which effectively slows down the chilling effect of the blank surface, and avoids the sudden drop of material fluidity and the sharp increase in deformation resistance caused by temperature drop that is too fast. The reduction in the maximum principal stress directly reduces the cracking tendency of the material under tensile stress, so the damage value also decreases significantly. For the surface quality and strain uniformity, the chilling effect not only increases the stress, but also causes the surface metal of the billet to be difficult to deform, resulting in defects such as folding or insufficient filling on the forging surface. The die preheating ensures the plasticity of the surface metal, so that the metal can fill the die cavity smoothly and completely, so as to obtain joint forgings with good surface quality. At the same time, the coordination of the overall deformation is improved, and the strain uniformity index increases, as shown in Table 4. In order to verify the reliability of the simulation calculation, the pilot test was used to carry out the warm extrusion forming of the drill pipe joint with the die at 300 °C. The warm extrusion can not only form good metal fibers, but also save a lot of metal raw materials.

### 4.3. Experimental Verification Analysis

**Figure 10:**
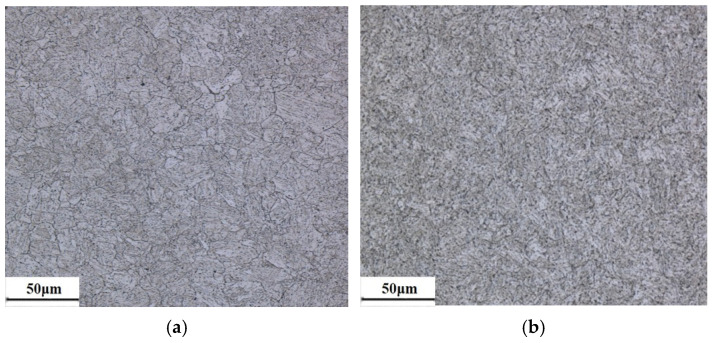
Microstructure of drill pipe joint after hot forming: (**a**) poor process control, (**b**) precise process control.

The microstructure under poor process control is shown in Figure 10a, forming under 1000 °C billet temperature, 20 mm/s punch speed and 20 °C die temperature; it is then water quenched after holding at 890 °C for 50 min, and then tempered at 450 °C for 120 min, which exhibits uneven grain size and mixed microstructural morphology; such microstructures often accompany anisotropic mechanical properties and insufficient toughness reserves. After undergoing “customized microstructure” precision hot forming–quenching and tempering process control, forming under 880 °C billet temperature, 20 mm/s punch speed and 300 °C die temperature, water is quenched after holding at 890 °C for 50 min, and then tempered at 450 °C for 120 min; the joint microstructure (Figure 10b) is transformed into a fine and uniform tempered sorbite structure (grain size up to grade 10.0), with highly refined microstructural morphology and uniform distribution, effectively eliminating the coarse and uneven nature and directional segregation of the original microstructure. Therefore, the control effect of forming temperature on microstructure is very significant: under the forming conditions of 1000 °C billet and 20 °C mold, there is intense heat exchange between the billet and the cold mold, and a significant quenching effect occurs on the surface of the billet, resulting in its actual deformation temperature being significantly lower than the initial molding temperature.

The forming conditions of 880 °C billet and 300 °C mold greatly alleviate surface quenching, making the plastic deformation and dynamic recovery/recrystallization of the material more uniform during the deformation process, and ultimately resulting in a more uniform distribution of microstructure and grains. This indicates that reasonable matching of billet and mold temperatures can improve the temperature field uniformity during the forming process, weaken the genetic effect of microstructure during deformation, and provide a more uniform microstructure basis for subsequent quenching and tempering treatment, thus obtaining a more uniform tempered martensite microstructure. This significant improvement in microstructure directly verifies the effectiveness of the technical path of “precise control of thermal process parameters—regulation of plastic-forming process—optimization of quenching and tempering heat treatment”, providing a reliable microstructural guarantee for the joint to simultaneously achieve high strength, high toughness, and low anisotropy. Under the optimal forming process parameters, the yield strength is 950 MPa, tensile strength is 1070 MPa, the impact energy at 20 °C is 123 J, and the hardness is 35 HRC.

This paper clarifies the regulation mechanism of temperature field and strain field on austenitization, phase transformation, and texture evolution during the thermal process, rather than merely staying at the simple correspondence of “parameters–properties”. Through finite element simulation, the impact of process fluctuations (such as temperature deviation and forming rate fluctuation) on microstructural uniformity is quantified, and the acceptable range of process fluctuations is clarified, providing a fault-tolerant space for stability control in industrial production. Currently, the relevant simulation models and process parameters can be directly applied to the hot forming and quenching and tempering process optimization of drill pipe joints, providing a directly reusable parameter control scheme for industrial production. This can effectively improve the first-pass yield and performance stability of the joints, reducing the risk of early failure caused by uneven microstructure.

## 5. Conclusions

(1) For a new high-strength high-toughness low-carbon steel used in 130 ksi drill pipe joints, controlled plastic deformation in the austenite recrystallization zone creates a mixed structure of high dislocation density, fine substructure, and fine grains. This provides abundant nucleation sites for martensite, yielding finer and more uniform transformation products.

(2) By adjusting deformation temperature and strain rate, a balance between dynamic recovery and recrystallization is achieved. At 1000–1100 °C and 0.1–1 s^−1^, small- and large-angle grain boundaries work together to refine grains, inhibit strong texture, and ensure uniform microstructure with low anisotropy.

(3) Optimal hot forming parameters for 130 ksi drill pipe joints are blank 880 °C, die 300 °C, and punch speed 20 mm/s, which reduce deformation resistance and damage while enhancing metal flow and strain uniformity. After quenching and tempering, the joint achieves a fine, uniform tempered sorbite structure (grain size 10.0) and optimal comprehensive strength and toughness.

## Figures and Tables

**Table 1:** Chemical composition analysis of 130 ksi steel (wt.%).

C	Si	Mn	Cr	Mo	Ni	Nb	V	P	S	Fe
0.08	0.29	1.75	1.05	0.5	0.2	0.05	0.2	0.008	0.003	Bal.

**Table 2:** Peak stress under different deformation conditions (MPa).

$\dot{\varepsilon }/s^{-1}$	T/°C			
	900	1000	1100	1200
0.01	75.69	51.66	42.1	44.74
0.1	135.19	91.26	59.57	65.75
1	142.83	117.43	86.3	78.41
10	202.7	142.75	125.76	89.16

**Table 3:** Field variables at different loading speeds.

Loading Stage	Loading Speed/mm/s	Equivalent Stress/MPa	Maximum Principal Stress/MPa	Strain Uniformity Index	Maximum Rate/mm/s	Damage Value
The first stage	1	397	199	0.50	20.4	0.834
	10	378	136	0.50	222	1.02
	20	387	106	0.50	407	0.659
The second stage	1	565	268	0.51	1.07	1.72
	10	557	245	0.51	22.1	1.42
	20	495	303	0.51	40	0.698

**Table 4:** Field variables at different mold temperatures.

Loading Stage	Loading Speed/mm/s	Equivalent Stress/MPa	Maximum Principal Stress/MPa	Strain Uniformity Index	Maximum Rate/mm/s	Damage Value
The first stage	20	378	136	0.50	222	1.02
	300	368	272	0.51	127	0.513
The second stage	20	557	245	0.51	22.1	1.42
	300	447	234	0.52	24.0	0.621

## Data Availability

The original contributions presented in this study are included in the article. Further inquiries can be directed to the corresponding authors.
